# Hemoglobin and hematocrit levels are positively associated with blood pressure in children and adolescents 10 to 18 years old

**DOI:** 10.1038/s41598-021-98472-0

**Published:** 2021-09-24

**Authors:** Hwal Rim Jeong, Young Suk Shim, Hae Sang Lee, Jin Soon Hwang

**Affiliations:** 1 Department of Pediatrics, College of Medicine, Soonchunhyang University, Cheonan Hospital, Cheonan-si, Korea; 2 Department of Pediatrics, Ajou University Hospital, Ajou University School of Medicine, 164 World cup-ro, Yeongtong-gu, Suwon, 16499 Korea

**Keywords:** Paediatric research, Paediatric research

## Abstract

To investigate the associations between hemoglobin (Hb) concentration and hematocrit (Hct), and blood pressure (BP) in children and adolescents. The study population consisted of 7950 subjects total (4229 boys and 3721 girls) aged 10–18 years who participated in the Korea National Health and Nutrition Examination Surveys conducted between 2007 and 2017. The prevalence of hypertension was 19.19% (21.51% for boys and 16.5% for girls) among the study population, and the prevalence of obesity was 9.59% (10.5% for boys and 8.6% for girls). Hb count and Hct tended to increase with the degree of obesity and BP elevation. Systolic BP (SBP) and diastolic BP (DBP) positively correlated with Hb count and Hct in both sexes. Following multiple linear regression analysis, Hb count and Hct presented a positive association with SBP and DBP after adjusting for age, BMI SDS, alcohol consumption, smoking status, physical activity, rural residence, household income, diagnosis of T2DM, hypertension, and dyslipidemia. Hb count and Hct were positively associated with SBP and DBP in children and adolescents 10–18 years old.

## Introduction

Hypertension (HTN) is an important risk factor for cardiovascular disease^1^ and a large contributor to the global burden of disease^2,3^. HTN in childhood often leads to HTN in adulthood, as childhood BP predicts adult BP^4–7^. It is a growing health problem that is often overlooked^8,9^.

In children younger than 13 years old, elevated blood pressure (BP) is defined as BP in the 90th percentile or higher considering age, height, and sex, and HTN is defined as BP in the 95th percentile or higher^10^. In adolescents 13 years or older, elevated BP is defined as 120 to 129 mm Hg systolic BP and less than 80 mm Hg diastolic BP, and HTN is defined by a BP of 130/80 mm Hg or higher^10^. HTN in childhood may contribute to premature atherosclerosis and the early development of cardiovascular disease (CVD). Therefore, identifying the prevalence of HTN and determining its potential risk factors in children and adolescents would be useful for understanding its pathophysiology and improving its treatment and prevention. Obesity is known as a risk factor for childhood HTN^11^; in turn, childhood HTN is increasing alongside obesity prevalence as it reaches epidemic proportions worldwide. Chronic kidney disease and sleep disorders are also attributed to the development of HTN^12,13^. Family history of HTN or CVD, male sex, low birth weight, and maternal smoking during pregnancy are additional risk factors, whereas children who were breastfed have a reduced risk for HTN^14,15^. Also, the risk of HTN varies according to race and ethnicity; Hispanic and black children are known to have a greater risk^16^.

Abnormalities in blood viscosity have been implicated in a number of CVDs^17,18^. Hemoglobin (Hb) is the most important determinant of whole blood viscosity^19^, and high Hb levels could be a potential risk factor to increase BP. Several studies have reported that Hb concentrations are elevated in humans with HTN. However, only a limited number of large-population studies have shown an association between Hb concentration and BP^20–23^. Also, such population studies conducted in childhood are rare. Therefore, the current study investigated the prevalence of HTN and obesity and the associations between Hb count and hematocrit (Hct), and BP in children and adolescents using data from the 2007–2017 Korea National Health and Nutrition Examination Survey (KNHANES).

## Results

A total of 7950 subjects were included in the final analysis. Among them, 4229 were boys (53.2%), and 3721 were girls. The subjects were categorized into three groups according to body mass index (BMI, kg/m^2^); 3343 of the boys were normal weight (NW, 79.0%), 443 were overweight (OW, 10.5%), and 443 were obese (OB, 10.5%), and 3059 of the girls were normal weight (82.2%), 342 were overweight (9.2%), and 320 were obese (8.6%).

**Figure 1:**
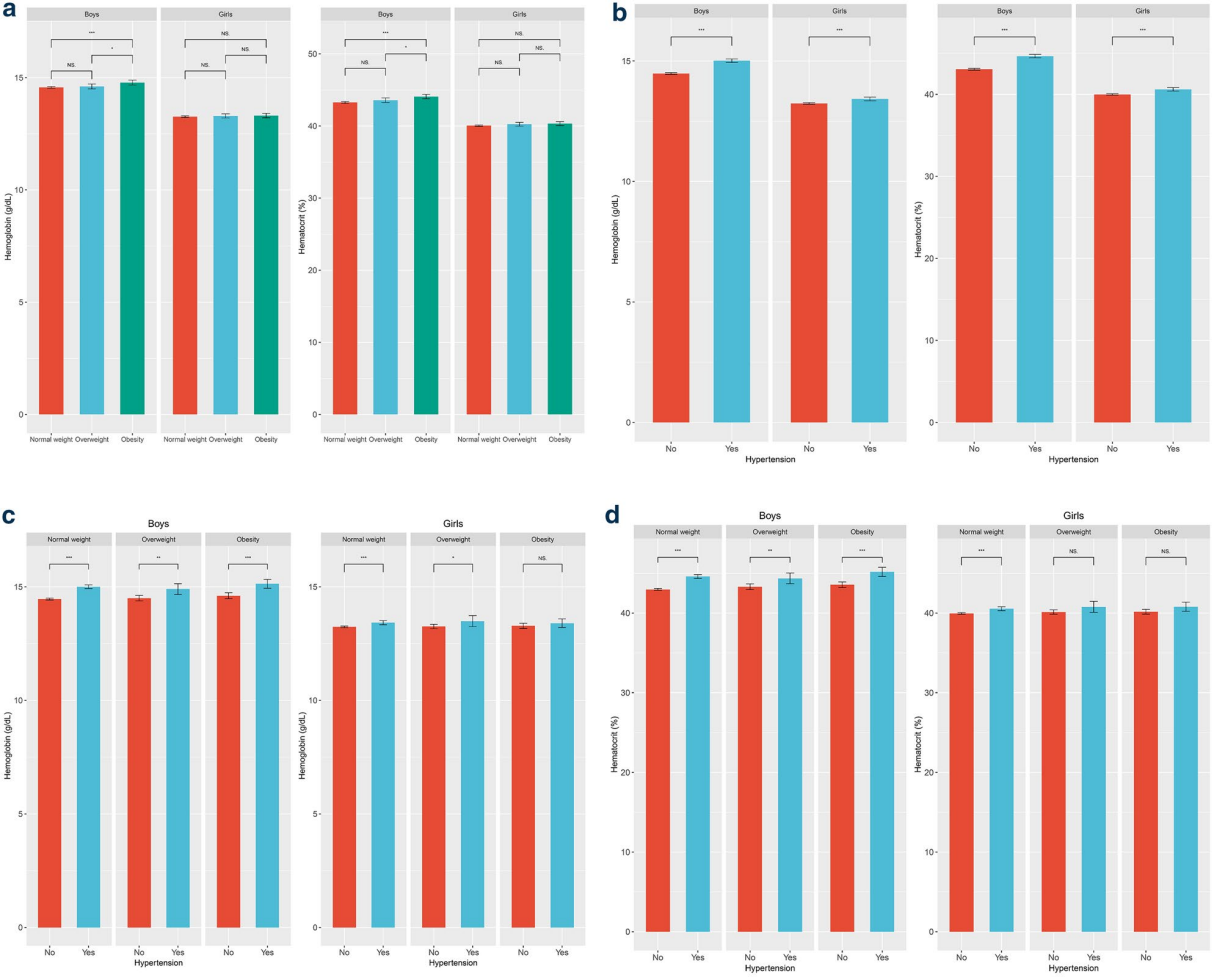
(**a**) Hemoglobin count and hematocrit according to BMI group in boys and girls. *p < 0.05,***p < 0.001. (**b**). Hemoglobin count and hematocrit according to hypertension status in boys and girls. ***p < 0.001. (**c**). Hemoglobin count according to BMI group, and hypertension status in boys and girls. *p < 0.05, **p < 0.005, ***p < 0.001, NS, *p* > 0.05. (**d**). Hematocrit according to BMI group, and hypertension status in boys and girls. *p < 0.05; **p < 0.005; ***p < 0.001; NS, *p* > 0.05.

Table 1 presents the clinical characteristics of the study population. The SDSs of height and waist circumference were significantly higher for boys than for girls. Systolic BP (SBP), diastolic BP (DBP), and serum glucose concentrations were higher for boys, and concentrations of TC, TG, HDL-C, and LDL-C were higher for girls. Alcohol consumption, smoking, and physical activity were more common in boys. None of the participants were previously diagnosed with HTN. However, this study has shown that 1526 adolescents did, in fact, have HTN (910 boys and 616 girls). The prevalence of HTN in childhood (10–18 years) was about 19.19% (21.5% for boys and 16.5% for girls). In the NW group, the prevalence of HTN was about 19.3% in boys and 15.2% in girls. In the OW group, the prevalence of HTN was about 26.1% in boys and 18.7% in girls. In the OB group, the prevalence of HTN was about 32.9% in boys and 26.8% in girls. Mean Hb count (g/dL) was 14.59 ± 1.13 in boys and 13.27 ± 0.94 in girls, and mean Hct (%) was 43.40 ± 3.33 in boys and 40.09 ± 2.56 in girls. Figure 1a reveals mean Hb count and Hct according to BMI group. Mean Hb and Hct increased significantly in boys between NW, OW, and OB groups. The mean Hb count (g/dL) was 14.57 ± 1.11 (NW), 14.61 ± 1.16 (OW), and 14.78 ± 0.01 (OB) in boys (*p* < 0.001), and the mean Hct (%) was 43.28 ± 3.31(NW), 43.58 ± 3.41(OW), and 44.09 ± 3.30 (OB) in boys (*p* < 0.001). The mean Hb count (g/dL) was 13.27 ± 0.95(NW), 13.30 ± 0.85(OW), and 13.31 ± 0.89 (OB) in girls (*p* = 0.587). Mean Hct (%) was 40.05 ± 2.58 (NW), 40.25 ± 2.44 (OW), and 40.34 ± 2.43 (OB) in girls (*p* = 0.079). Mean Hb count and Hct tended to increase among BMI groups, but there was no statistical significance in girls.

**Table 1:** Clinical characteristics of the study population (*n* = 7950).

	Boys	Girls	*p*
	(*n* = 4229)	(*n* = 3721)	
Age (years)	14.31 ± 2.51	14.37 ± 2.51	0.319
Height SDS	0.25 ± 1.05	0.19 ± 1.05	0.008
Weight SDS	0.10 ± 1.22	0.06 ± 1.14	0.102
BMI SDS (kg/m^2^)	− 0.25 ± 1.14	− 0.19 ± 1.09	0.656
WC SDS	− 0.04 ± 1.29	− 0.05 ± 1.19	0.022
SBP (mmHg)	108.79 ± 10.60	104.23 ± 9.32	< 0.001
DBP (mmHg)	66.48 ± 9.45	65.59 ± 8.26	< 0.001
Hemoglobin (g/dL)	14.59 ± 1.13	13.27 ± 0.94	< 0.001
Hematocrit (%)	43.40 ± 3.33	40.09 ± 2.56	< 0.001
Glucose (mg/dL)	90.78 ± 7.70	89.34 ± 8.09	< 0.001
T-C (mg/dL)	155.83 ± 27.11	163.79 ± 26.23	< 0.001
HDL-C (mg/dL)	49.84 ± 9.90	52.22 ± 9.85	< 0.001
TGs (mg/dL)	82.90 ± 47.32	86.50 ± 44.16	0.001
LDL-C (mg/dL)	89.40 ± 23.28	94.27 ± 22.84	< 0.001
Alcohol drinker	1155 (27.31%)	855 (22.98%)	< 0.001
Smoker	668 (15.80%)	245 (6.58%)	< 0.001
Household income ≤ 1st quartile	460 (10.88%)	410 (11.02%)	0.869
Rural residence	689 (16.29%)	605 (16.26%)	0.992
Physical activity, yes/no	2474/1755 (58.50%)	2052/1669 (55.15%)	0.003
Diagnosis of hypertension	0	0	> 0.999
Diagnosis of T2DM	2 (0.05%)	1 (0.03%)	> 0.999
Diagnosis of dyslipidemia	0	0	> 0.999

*SDS* standard deviation score; *WC* waist circumference; *BMI* body mass index; *SBP* systolic blood pressure; *DBP* diastolic blood pressure; *T-C* total cholesterol; *HDL-C* high-density lipoprotein cholesterol; *TGs* triglycerides; *LDL-C* low-density lipoprotein cholesterol; *T2DM* type 2 diabetes mellitus.

Figure 1b reveals mean Hb count and Hct according to HTN status. The mean Hb count (g/dL) was significantly higher in subjects with HTN than in boys (14.90 ± 1.1 vs. 14.44 ± 1.0, *p* < 0.001) or girls (13.43 ± 0.93 vs. 13.21 ± 0.93, *p* < 0.001) with normal BP. Mean Hct (%) was also significantly higher in subjects with HTN than in boys (44.31 ± 3.28 vs. 42.95 ± 3.26, *p* < 0.001) or girls (40.55 ± 2.56 vs. 39.91 ± 2.53, *p* < 0.001) with normal BP.

Mean Hb count and Hct according to sex, BMI group, and presence of HTN are shown in Table 2 and Fig. 1c,d. In boys, when analyzed in all groups according to BMI group, Hb count and Hct were significantly higher in those with HTN than in those with normal BP (*p* < 0.001). In girls in the NW group, Hb count and Hct were significantly higher in those with HTN than in those with normal BP (*p* < 0.001). In girls in the OW group, Hb count was significantly higher in those with HTN (*p* < 0.05*)*, but Hct was not (*p* = 0.083). In girls in the OB group, Hb count and Hct were high in those with HTN, but there was no statistically significant difference (*p* = 0.295 and *p* = 0.054, respectively).

**Table 2:** Hemoglobin count and hematocrit according to sex, body mass index, and hypertension status.

	Subgroup n (%)	Boys (n = 4229)			Girls (n = 3721)		
		NW	OW	OB	NW	OW	OB
		3343 (79.0%)	443 (10.5%)	443 (10.5%)	3059 (82.2%)	342 (9.2%)	320 (8.6%)
	HTN diagnosis (%)	648 (19.3%)	116 (26.1%)	146 (32.9%)	466 (15.2%)	64 (18.7%)	86 (26.8%)
**Hb count (g/dL)**	Normal BP	14.46 ± 1.10	14.50 ± 1.07	14.61 ± 1.15	13.24 ± 0.94	13.25 ± 0.82	13.28 ± 0.90
	HTN	15.00 ± 1.05	14.90 ± 1.28	15.13 ± 1.19	13.49 ± 0.96	13.49 ± 0.96	13.40 ± 0.88
**Hct (%)**	Normal BP	42.97 ± 3.29	43.31 ± 3.28	43.56 ± 3.07	39.96 ± 2.56	40.13 ± 2.34	40.17 ± 2.32
	HTN	44.59 ± 3.05	44.36 ± 3.66	45.18 ± 3.50	40.55 ± 2.66	40.78 ± 2.76	40.80 ± 2.66

Data are presented as mean ± standard deviation.

*NW* normal weight; *OW* overweight; *OB* obese; *HTN* hypertension; *Hb* hemoglobin; *Hct* hematocrit; *BP* blood pressure.

HTN was defined as (i) systolic blood pressure (SBP) or diastolic blood pressure (DBP) ≥ the 95th percentile considering age, sex, and height or (ii) SBP ≥ 130 mm Hg or DBP ≥ 80 mm Hg.

Table 3 presents the correlation between Hb count and Hct, and BP in all subjects. Hb count and Hct were both positively correlated with SBP and DBP in boys and girls before and after adjusting for age.

**Table 3:** Unadjusted and adjusted correlations between hemoglobin count and hematocrit, and blood pressure in children and adolescents 10–18 years old (*n* = 7950).

	No of participants	Unadjusted		Adjusted for age	
		r	*p*	r	*p*
**Hb count (g/dL)**					
SBP (mmHg)					
Boys	4229	0.320	< 0.001	0.121	< 0.001
Girls	3721	0.042	0.010	0.058	< 0.001
DBP (mmHg)					
Boys	4229	0.341	< 0.001	0.119	< 0.001
Girls	3721	0.045	0.006	0.103	< 0.001
**Hct (%)**					
SBP (mmHg)					
Boys	4229	0.345	< 0.001	0.150	< 0.001
Girls	3721	0.098	< 0.001	0.103	< 0.001
DBP (mmHg)					
Boys	4229	0.339	< 0.001	0.114	< 0.001
Girls	3721	0.088	< 0.001	0.123	< 0.001

*Hb* hemoglobin; *Hct* hematocrit; *SBP* systolic blood pressure; *DBP* diastolic blood pressure.

Table 4 presents the multiple linear regression between Hb count and Hct, and BP in boys and girls. The mean SBP and DBP increased alongside Hb count and Hct in both sexes. Model 2 demonstrated that Hb count and Hct were significantly associated with BP after adjusting for age, BMI SDS, alcohol consumption, smoking status, physical activity, rural residence, household income, diagnoses of type 2 diabetes mellitus (T2DM), HTN, and dyslipidemia. In boys, SBP increased by 1.3 mm Hg with every 1 g/dL increase in Hb concentration. SBP in girls increased by 0.5 mm Hg with every 1 g/dL increase in Hb concentration. The DBP patterns were similar to the patterns observed with SBP. DBP increased by 1.1 mm Hg in boys and 0.8 mm Hg in girls with every 1 g/dL increase in Hb concentration. SBP increased by 0.5 mm Hg in boys and 0.3 mm Hg in girls with every 1% increase in Hct. DBP increased by 0.3 mm Hg with every 1% increase in Hct for both sexes.

**Table 4:** Multiple linear regression of hemoglobin count and hematocrit against blood pressure in children and adolescents 10–18 years old (*n* = 7950).

Variable	No. of participants	Model 1			Model 2		
		β	SE	*p*	β	SE	*p*
**Hb count (g/dL)**							
SBP (mmHg)							
Boys	4229	1.333	0.168	< 0.001	1.341	0.168	< 0.001
Girls	3721	0.570	0.161	< 0.001	0.553	0.161	< 0.001
DBP (mmHg)							
Boys	4229	1.201	0.155	< 0.001	1.196	0.155	< 0.001
Girls	3721	0.893	0.142	< 0.001	0.889	0.142	< 0.001
**Hct (%)**							
SBP (mmHg)							
Boys	4229	0.559	0.057	< 0.001	0.569	0.057	< 0.001
Girls	3721	0.366	0.058	< 0.001	0.351	0.058	< 0.001
DBP (mmHg)							
Boys	4229	0.390	0.052	< 0.001	0.389	0.053	< 0.001
Girls	3721	0.389	0.051	< 0.001	0.387	0.051	< 0.001

*Hb* hemoglobin; *Hct* hematocrit; *SBP* systolic blood pressure; *DBP* diastolic blood pressure; *BMI* body mass index; *SDS* standard deviation score; *T2DM* type 2 diabetes mellitus.

Model 1: Statistical significance was determined using multiple linear regression of Hb count and Hct versus blood pressure after adjusting for age and BMI SDS.

Model 2: Statistical significance was determined using multiple linear regression of Hb count and Hct versus blood pressure after adjusting for age, BMI SDS, alcohol consumption, smoking status, physical activity, rural residence, household income, diagnosis of T2DM, hypertension, and dyslipidemia.

## Discussion

This study showed the prevalence of obesity and HTN and revealed the positive associations between Hb count and hematocrit level, and BP in children and adolescents. To our knowledge, this is the first study that investigated the associations between Hb count and Hct, and systolic and diastolic BP in a large sample of Korean children and adolescents aged 10–18 years. Our study population was representative of Korean children and adolescents because the KNHANES is an official nationwide database that reflects geographic and demographic differences throughout Korea.

Recently, the prevalence of above-healthy BMI has increased rapidly in children and adolescents^24^. Obesity is a multifactorial disease caused by genetic, biological, environmental, behavioral, and psychological factors^25^. Childhood obesity leads to adult obesity^26,27^ and an increased risk for cardiovascular disease and mortality in adulthood^28^. Moreover, severe obesity in children is positively associated with cardiometabolic risk factors, including low HDL cholesterol, high SBP and DBP, high TG, and high glycated Hb levels^29^.

The prevalence of above-healthy BMI was reported to be more than 10% in developing countries and even higher (20%) in developed countries^30^. Reports on the prevalence of childhood obesity in Korea vary, but it has also increased over time. In 2013, the prevalence of overweight BMI in children younger than 20 years old per 100,000 children was 21.2% in boys and 13.2% in girls, and the prevalence of obesity was 4.8% in boys and 3.1% in girls^30^. In our study considering data from those aged 10–18 years collected between 2007 and 2017, the overall prevalence of overweight BMI was 21% in boys and 17.8% in girls, and the prevalence of obesity was 10.5% in boys and 9.2% in girls. Our study did not analyze the annual prevalence of obesity and overweight BMI, and the difference in prevalence between our study and the previous report was likely caused by the age difference between subjects and the method of analysis.

The prevalence of elevated BP and HTN in children is reported to be around 6% and 3%, respectively^9,31^. In obese adolescents, the combined prevalence increases to around 30%^31^. Cho H et al. reported that the prevalence of HTN has increased over time from 6.9% (KNHANES 2007–2009) to 9.0% (KNHANES 2013–2015) in Korean children and adolescents 10–18 years old^32^. In the previous study, the prevalence of HTN was higher in boys than in girls and increased alongside the degree of obesity. Especially in the obesity group, the prevalence of HTN was higher than the sample average. In our study, the prevalence of HTN was also higher in boys than in girls and increased alongside the severity of obesity. However, our study design and methodology are different than previous studies, and the current study reported the prevalence of HTN to be 19.2% sample-wide (KNHANES 2007–2017) and 30.4% in the obesity group. In our study, we evaluated BP according to reference BP assessments in Korean children, different from the American Academy of Pediatrics (AAP) guidelines. According to the AAP guideline^10^, HTN criteria in children ≥ 13 years old is ≥ 130/80 mm Hg. However, in the present study, HTN was defined as SBP or DBP in or above the 95th percentile in children ≥ 13 years old or BP ≥ 130/80 mm Hg based on the Korean guidelines^33^, which are considered to be lower than the AAP guidelines. Therefore, the prevalence of HTN in our study seems to be higher than in previous reports.

The results of our study show that the prevalences of obesity and HTN were higher in boys than girls, and the prevalence of HTN was higher in the obese and overweight groups than in the normal-weight group in both sexes. Hb count and Hct tended to increase with the degree of obesity and BP elevation in both sexes, but there some differences between sexes. In boys and girls, Hb count and Hct higher in the HTN group than in those with normal BP. In boys alone, Hb count and Hct were significantly elevated in the OB group compared to that of the NW and OW groups. On the other hand, in girls, Hb count and Hct were no different according to obesity. As previously mentioned, obesity is a known risk factor of HTN^11^. In our study, HTN is positively associated with Hb count and Hct. Although there were gender-related differences, obesity might be related to increased Hb count and Hct. Further study should be performed to verify it.

Several studies have investigated the associations between Hb concentration and Hct, and BP; however, most of such investigations are performed in adults^20–23^. Previous studies have reported increased Hb concentrations in those with HTN and positive associations between SBP and DBP, and Hb count. The results of our study are in line with such findings^20–23,34–36^. Atsma et al. reported that Hb level was positively associated with both SBP and DBP in healthy Dutch voluntary blood donors 18–70 years of age^21^. They found 1.3 mm Hg and 1.8 mm Hg increases in SBP with every 1 mmol/L increase in Hb count for men and women, respectively, and DBP rose 1.4 mm Hg and 1.5 mm Hg with every 1 mmol/L increase in Hb count for men and in women, respectively. Lee et al. reported that SBP in men with Hb concentrations ≥ 13.0 g/dL and in women with Hb concentrations ≥ 11.0 g/dL increased by 2.6 mm Hg for every 1 mmol/L increase in Hb concentration, and DBP increased by 3.2 mm Hg for every 1 mmol/L increase in Hb concentration for both Korean men and women aged ≥ 20 years^23^. In our study, the regression coefficients were relatively lower than the previous study. SBP increased by 1.3 mm Hg in boys and 0.5 mm Hg in girls with every 1 g/dL increase in Hb level, and DBP increased by 1.1 mm Hg in boys and 0.8 mm Hg in girls with every 1 g/dL increase in Hb level. In our study, subjects were 10–18 years old, younger than in previous studies, and the units for Hb count were g/dL instead of mmol/L, so the regression coefficient seemed to be smaller in comparison. It is estimated that the difference in study design, ethnicity, and characteristics of the study population may have caused the difference in results.

Göbel et al. found significant correlations between mean arterial BP and red blood cell count, Hct, and Hb concentration in healthy subjects. They suggested that Hct plays a role in determining the viscosity of the blood and that it may be involved as a rheological factor controlling BP^22^. Our study did not collect red blood cell counts but indicated that Hct and Hb level were involved in BP determination, although the mechanisms for BP elevation alongside Hb count and Hct are not entirely known. Previous research has demonstrated Hct levels to be positively associated with HTN incidence^37^. Furthermore, increased Hct count is associated with increased blood viscosity, and increased viscosity is considered a determinant of vascular resistance, expected to contribute to BP^38,39^. The fact that polycythemia vera is often associated with cardiovascular disease supported this theory as plausible^40^. Moreover, Kawamoto reported that Hb count is strongly related to arterial stiffness, which, in turn, increases SBP and DBP^41^. Therefore, they suggested that slightly low Hb levels were beneficially associated with arterial stiffness in women. In addition, Cabrales et al*.* reported that Hb is a scavenger of nitric oxide (NO), produced in endothelial cells^42^. NO acts as a vasodilator and to prevent the development of atherosclerosis and its complications^43^. Increased levels of free Hb induce vasoconstriction due to NO scavenging and consequently impart BP elevation.

The current study has some limitations. First, since our study is cross-sectional, evaluation of causality was not possible. Second, while the prevalence of HTN was approximated using a population sample, this approximation may be flawed due to the sample’s likely imperfect representation of Korea’s demographics. The BP standard data for Korean children also differs from AAP guidelines. Therefore, the prevalence of HTN in our study may have been overestimated compared to if the AAP guidelines were used. Also, white coat HTN was not excluded in our study. Third, unweighted prevalence using conventional analysis can differ from weighted prevalence using survey analysis, as previous Korean studies have reported weighted prevalence that was not observed in the present study^32^. Nevertheless, both survey analysis for weighted prevalence and the conventional method for unweighted prevalence showed similar results for overall prevalence of hypertension (19.9% weighted prevalence for hypertension and 19.2% unweighted prevalence for hypertension) (Supplementary Table 1) and similar patterns of changes in prevalence for hypertension according to sex and year (Supplementary Figs. 1) in further analyses. Finally, the cause of the sexual-dependent relationships between Hb count and Hct, and BP were not fully elucidated. Periodic menstrual bleeding in girls might have influenced the difference in results. However, we had no data regarding pubertal status, diagnosis of anemia, or menstrual loss in the present study. Subsequent prospective studies are needed to clarify the mechanisms by which BP increases alongside Hb count and Hct and to determine the cause of the discrepancy between sexes.

In conclusion, Hb count and Hct were positively associated with SBP and DBP in Korean children and adolescents aged 10–18 years. Both Hb count and Hct contribute to BP, and their increase could be a potential risk factor for high BP. In particular, obesity could have a synergistic effect with Hct and Hb count to further elevate BP. It is important to identify and manage the risk factors for cardiovascular disease from childhood and adolescence. Establishing the CVD risk factors and their management would be the cornerstone of long-term healthcare that influences lifelong well-being. Further research is required to identify and verify the factors that increase BP in children of all ages, including newborns and other ethnic groups.

## Material and methods

Data from the 2007–2017 KNHANES were analyzed in this study. The survey has been conducted by the Division of Chronic Disease Surveillance in the Korean Centers for Disease Control and Prevention on a 3-year cycle since 1998 to assess the health and nutritional status of the noninstitutionalized civilian population of Korea^44^. The KNHANES is a cross-sectional and nationally representative survey with a multistage and stratified probability sampling design. Data acquired from the full fourth (2007–2009), fifth (2010–2012), and sixth (2013–2015) cycles and the first year of the seventh cycle (2017) were combined to enhance the statistical power of this analysis.

A total of 89,630 individuals were included. Of these subjects, 7950 participants aged 10–18 years were included in the preliminary analysis. All subjects and their parents were interviewed at home after providing informed consent and underwent various examinations, including blood sampling^45^. Those with incomplete records regarding physical examination, including anthropometric measurements, BP, and laboratory tests, such as the lipid profile, were excluded. Those who had serum TGs ≥ 400 mg/dL were also excluded (n = 16). The database is available to the public at the KNHANES website (http://knhabes.cdc.go.kr). The study protocols of the 2007–2017 KNHANES survey were approved by the institutional review boards of the Korean Centers for Disease Control and Prevention. Informed consent was provided by all KNHANES subjects. All experiments were performed in accordance with relevant guidelines and regulations.

Anthropometric assessments, including height, weight, WC, SBP, and DBP, were performed by a trained expert. Height was measured to the nearest 0.1 cm using an electronic stadiometer (SECA, Germany). Weight was measured to the nearest 0.1 kg with an electronic scale (G-TECH, Korea). WC was measured to the nearest 0.1 cm using a calibrated measuring tape (SECA). SBP and DBP were measured 3 times to the nearest 1 mm Hg using a standard mercury sphygmomanometer. The SD scores (SDS) for height, weight, WC, and BMI were calculated using age- and sex-specific least mean square (LMS) parameters based on the 2017 growth reference values for Korean children and adolescents developed by the Korean Pediatric Society and the Korea Centers for Disease Control and Prevention^46^. The subjects were categorized into 3 groups according to BMI: normal weight (NW, BMI < 85th percentile), overweight (OW, BMI between the 85th percentile and the 95th percentile), and obese (OB, BMI ≥ 95th percentile). HTN was defined as sex-, age-, and height-specific SBP or DBP equal to or greater than the 95th percentile based on the Korean reference^33^, SBP ≥ 130 mmHg, or DBP ≥ 80 mmHg.

Lifestyle-related behaviors, such as alcohol consumption, smoking, household income, and residence, were assessed by means of a questionnaire. Information about alcohol consumption and smoking status (positive vs. negative current statuses) was collected with a self-administered questionnaire from subjects aged 12 years and older^45^. Physical activity was used to divide subjects into two groups (yes or no), and subjects were included in the ‘yes’ group if they performed intense physical activity ≥ 20 min/day ≥ 3 days/week, performed moderate physical activity ≥ 30 min/day ≥ 5 days/week, or if they walked ≥ 30 min/day ≥ 5 days/week.

Questionnaires on household income and area of residence (urban vs. rural) were administered by trained interviewers. Diagnoses of HTN, diabetes, and dyslipidemia were also included in the questionnaire.

### Statistical analyses

R, version 3.5.1 (The R Foundation for Statistical Computing, Austria), was used for statistical analysis. Continuous variables are expressed as mean ± SD. Categorical variables are presented as number and percentage. A two-tailed *t*-test was performed to identify differences in clinical parameters between boys and girls, whereas categorical variables were compared using the χ2 test. To determine statistical significance, we used analysis of variance (ANOVA) for Hb count and Hct according to BMI groups. The Pearson’s correlation coefficient analysis was performed to evaluate the correlations between Hb count and Hct, and BP. Multiple linear regression was performed to investigate the relationships between Hb count and Hct, and BP. Probability values less than 0.05 were considered statistically significant.

## Supplementary Information

Supplementary Information.
