# Supplementary material for: Hemoglobin and hematocrit levels are positively associated with blood pressure in children and adolescents 10 to 18 years old
Source: Sci Rep. 2021 Sep 24;11:19052. doi: 10.1038/s41598-021-98472-0 (PMC8463603; doi:10.1038/s41598-021-98472-0)

**Supplementary Files**

**Supplementary Table 1.** Changes in unweighted and weighted prevalence for hypertension according to sex and year.

|  | Unweighted prevalence | | |  | Weighted prevalence | | | |
| --- | --- | --- | --- | --- | --- | --- | --- | --- |
| Year | Total | Boys | Girls |  | Total | Boys |  | Girls |
| 2007 | 19.9% | 23.8% | 15.3% |  | 18.8% | 23.8% |  | 13.0% |
| 2008 | 15.8% | 19.9% | 11.0% |  | 17.9% | 23.0% |  | 12.1% |
| 2009 | 24.6% | 27.9% | 21.1% |  | 25.2% | 29.8% |  | 20.1% |
| 2010 | 19.5% | 22.8% | 15.6% |  | 20.8% | 24.6% |  | 16.3% |
| 2011 | 16.7% | 19.5% | 13.5% |  | 18.9% | 21.2% |  | 16.2% |
| 2012 | 19.5% | 19.3% | 19.8% |  | 19.7% | 20.7% |  | 18.4% |
| 2013 | 16.9% | 16.8% | 17.1% |  | 17.4% | 18.2% |  | 16.5% |
| 2014 | 18.0% | 19.8% | 15.9% |  | 17.0% | 19.0% |  | 14.7% |
| 2015 | 19.4% | 19.8% | 18.9% |  | 20.1% | 21.8% |  | 18.2% |
| 2016 | 20.3% | 23.5% | 16.7% |  | 20.9% | 24.4% |  | 17.1% |
| 2017 | 19.3% | 21.0% | 17.4% |  | 21.0% | 23.8% |  | 18.0% |
| Overall | 19.2% | 21.5% | 16.6% |  | 19.9% | 21.6% |  | 16.5% |

**Supplementary Figure 1.** Changes in unweighted and weighted prevalence for hypertension according to sex and year.


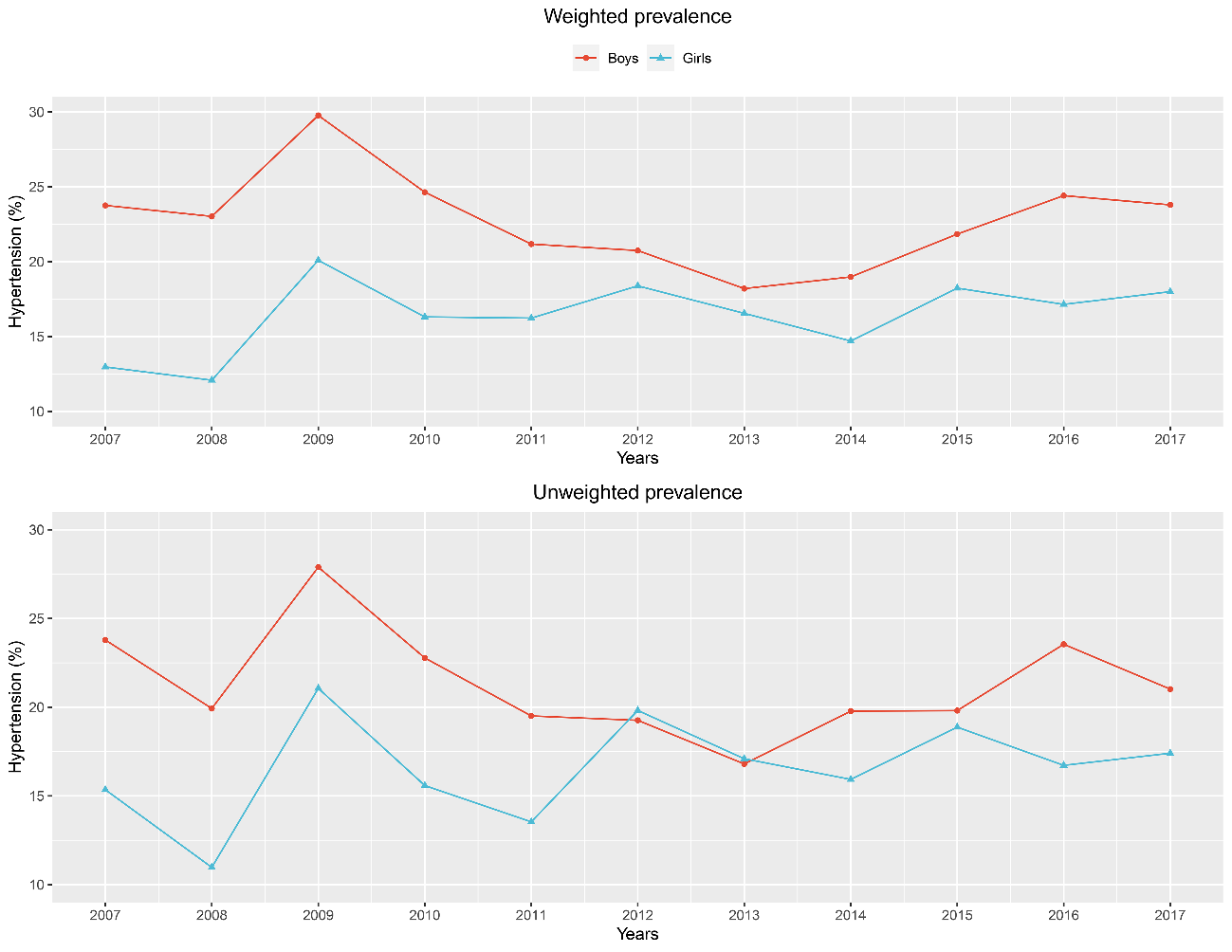

Supplement: Supplementary file 1 — Supplementary Information. [file 41598_2021_98472_MOESM1_ESM.docx]
